# Evaluating Seagrass Meadow Dynamics by Integrating Field-Based and Remote Sensing Techniques

**DOI:** 10.3390/plants11091196

**Published:** 2022-04-28

**Authors:** Danijel Ivajnšič, Martina Orlando-Bonaca, Daša Donša, Veno Jaša Grujić, Domen Trkov, Borut Mavrič, Lovrenc Lipej

**Affiliations:** 1 Faculty of Arts, University of Maribor, Koroška cesta 160, 2000 Maribor, Slovenia; dani.ivajnsic@um.si; 2 Faculty of Natural Sciences and Mathematics, University of Maribor, Koroška cesta 160, 2000 Maribor, Slovenia; dasa.donsa1@um.si; 3 Marine Biology Station Piran, National Institute of Biology, Fornače 41, 6330 Piran, Slovenia; martina.orlando@nib.si (M.O.-B.); domen.trkov@nib.si (D.T.); borut.mavric@nib.si (B.M.); lovrenc.lipej@nib.si (L.L.); 4 Faculty of Education, University of Maribor, Koroška cesta 160, 2000 Maribor, Slovenia

**Keywords:** Adriatic Sea, change analysis, *Cimodocea nodosa*, image classifiers, Sentinel-2

## Abstract

Marine phanerogams are considered biological sentinels or indicators since any modification in seagrass meadow distribution and coverage signals negative changes in the marine environment. In recent decades, seagrass meadows have undergone global losses at accelerating rates, and almost one-third of their coverage has disappeared globally. This study focused on the dynamics of seagrass meadows in the northern Adriatic Sea, which is one of the most anthropogenically affected areas in the Mediterranean Sea. Seagrass distribution data and remote sensing products were utilized to identify the stable and dynamic parts of the seagrass ecosystem. Different seagrass species could not be distinguished with the Sentinel-2 (BOA) satellite image. However, results revealed a generally stable seagrass meadow (283.5 Ha) but, on the other hand, a stochastic behavior in seagrass meadow retraction (90.8 Ha) linked to local environmental processes associated with anthropogenic activities or climate change. If systemized, this proposed approach to monitoring seagrass meadow dynamics could be developed as a spatial decision support system for the entire Mediterranean basin. Such a tool could serve as a key element for decision makers in marine protected areas and would potentially support more effective conservation and management actions in these highly productive and important environments.

## 1. Introduction

Seagrass meadows rank among the most productive and valuable ecosystems in the biosphere [1,2,3] and are therefore recognized as priority habitats in many environmental legislation and regulation, including the EU Habitat Directive (HD, 92/43/EEC). Over recent decades, seagrass meadows have undergone global losses at accelerating rates of 2–5% year^−1^ [2,4,5,6]. Almost one-third of their coverage globally has disappeared [7,8]. They are considered to be among the most threatened ecosystems on Earth, and their deterioration seriously affects their importance in terms of the ecosystem services they provide to coastal zones [9,10]. Moreover, they are considered crucial habitats for numerous invertebrates and fish, since they provide important feeding grounds, shelter and nursery habitat, even for many high-value commercial species [11,12,13]; they stabilize sediments [14,15], provide protection against coastal erosion [16], produce oxygen [17] and are recognized as global carbon sinks [18]. The loss of seagrass meadows results from a wide variety of causes, attributed to various factors, among which the most important are reduced water quality and eutrophication [19], deposition of sediments, extreme temperature related stress, overgrazing [20], and physical damage due to anchoring and dredging [21]. Diseases caused by various microbes have also been found to cause major seagrass disappearance [22,23]. There is also evidence that seagrass meadows are susceptible to climate change, which is expected to further accelerate seagrass decline in the near future on the local, regional and global scale [7,8,24,25,26]. 

In European marine waters, four native species of seagrass are present: *Posidonia oceanica* (Linnaeus) Delile, *Cymodocea nodosa* (Ucria) Ascherson, *Zostera marina* Linnaeus and *Zostera noltei* Hornemann [27]. All four are present along the northern Adriatic coastline, where *C. nodosa* is the most abundant [28]. *C. nodosa* is a common and widely distributed species in the whole Mediterranean Sea [29], forming extensive monospecific seagrass meadows or mixed meadows [30]. It can be found in coastal waters, coastal lagoons, inlets and estuaries and other sheltered and semi-exposed habitats [31,32]. Its distribution is mainly related to light availability and water depth [33]. *C. nodosa* is considered an indicator of environmental changes in the Mediterranean basin because of its widespread distribution, sensitivity to a variety of natural and anthropogenic impacts, and measurability of species response to those impacts [34]. Following Marine Strategy Framework Directive (MSFD, 2008/56/EC) requirements, the environmental status (EnS) of *C. nodosa* meadows was recently evaluated on the basis of leaf length in the northern Adriatic, mostly as high/good [32], but for this assessment, the meadows extent and stability were not taken into consideration. The proper assessment of the status of these meadows is of crucial importance for the assessment of the EnS according to Descriptors D1 (Biodiversity), D5 (Eutrophication) and D6 (Sea-floor integrity) and should include parameters like extent/coverage and stability/dynamics. Mapping and monitoring the coverage of *C. nodosa* meadows over time are prerequisites for the assessment of their status in different areas [35] and crucial for the interpretation of changes occurring in these important habitats and their biodiversity [36]. However, only limited studies dealing with long-term data about the distribution and coverage of seagrass meadows are available. This is mainly due to the lack of long-term data series about seagrass meadow extent. The same is true for the northern Adriatic Sea, where it is therefore currently difficult to assess the actual extent of the deterioration and loss of these crucial habitats, in order to support conservation action for seagrass meadows. The decline of *C. nodosa* seagrass meadows was recently reported in the southern Adriatic Sea. Mačić and Zordan [37] pointed out a 20% loss of *C. nodosa* coverage in Kotor Bay (Montenegro). 

With combined methodologies, the assessment of seagrass extent and dynamics should drastically improve, and the problem of data deficit could thus be eradicated. Nowadays, high spatial and temporal resolution satellite remote sensing data and advanced image processing techniques, driven by machine learning algorithms [38,39], offer a valuable approach for mapping seagrass meadow dynamics in shallow waters [35,40,41]. The Sentinel-2 twin polar-orbiting satellite mission (S2A and S2B), launched under the European Commission’s Copernicus program in 2015 [42,43], overcame several previously common issues in seagrass meadow remote sensing (intra-pixel mixture with other cover types, insufficient satellite revisit time and consequent problems with cloud cover, limitations linked to noise in the satellite data caused by scattering, reflection and absorption of light in the atmosphere, air-water interface, and water column) and thus enabled new mapping possibilities in shallow water environments [42,44,45,46]. However, since satellite image classification quality is highly dependent on good ground control samples, in situ sampling, or better, mapping remains a key segment in such studies. Indeed, both methodological approaches and types of data were utilized in this study to test the applicability of Sentinel-2 (BOA) satellite data for seagrass detection, to accurately evaluate past and current *C. nodosa* dominated seagrass meadow extent along the Slovenian coast, and finally, to identify the stable and dynamic parts of this valuable habitat. 

## 2. Results

### 2.1. The Field-Based Data Perspective

**Figure 1:**
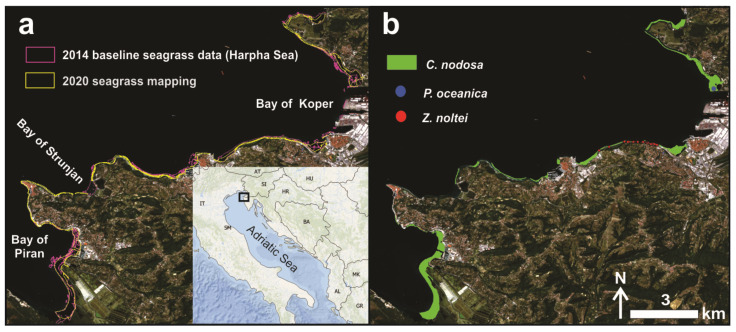
The 2014 baseline seagrass status and the 2020 mapping in the study area (**a**). Spatial distribution of *C. nodosa*, *P. oceanica* and *Z. noltei* (**b**).

The 2014 mapping revealed that seagrasses (*Cymodocea nodosa*, *Posidonia oceanica* and *Zostera noltei*) covered 282.4 Ha of area along the Slovenian coast. Typically, seagrass meadows thrive here on sedimentary (sandy-muddy) seafloor, at depths between 1 and 11 m. Results in 2020, where 265.1 Ha of the shallow sea between 1 and 4 m depth was again scanned, indicated seagrass meadow persistence on 148.1 Ha of area (only 0.64 Ha belong to *P. oceanica*, the rest to *C. nodosa* meadows, and very small patches to *Z. noltei*). Nowadays, the only remaining meadow of *P. oceanica* in the Gulf of Trieste has a patchy distribution, restricted to a depth range of 1 to 4 m, along an exposed shoreline (Figure 1b). *C. nodosa* is the most common species in shallow, sheltered to semi-exposed sites, and the surveyed meadows can be considered part of the biocoenosis of superficial muddy sands in sheltered waters. It also inhabits the lagoonary environment of the euryhaline and eurytherm biocoenosis and in the biocoenosis of well sorted fine sands. Such meadows are both monospecific and mixed with *Z. noltei*. Near the Port of Koper, there is a small monospecific seagrass meadow of *Z. noltei*, growing on the muddy bottom (Figure 1b). 

However, deeper waters were not included in the 2020 mapping, which could affect the overall estimation of spatio-temporal dynamics of seagrass meadows in the study area between 2014 and 2020. The lack of seagrass meadow distribution data in the lower infralittoral belt in 2020 was bridged by relying on satellite remote sensing technique, which are known to provide adequate results for shallow water habitats, particularly seagrasses. The cloud-free Sentinel-2 BOA product for 8 August 2020 (Figure 1A) was selected for this purpose, since it had the best water surface conditions (low wind speed) compared to other available cloud-free imagery during the target season.

### 2.2. Pre-Image Classification Procedures

**Figure 2:**
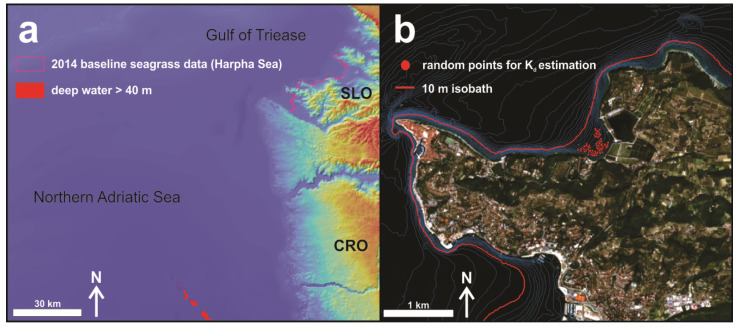
Identified pixels for infinitely deep-water reflectance estimation (**a**) and the location of random points for diffuse attenuation coefficients determination (**b**).

The first step was to provide missing data for Equation (1) and calculate the water column correction. Two bathymetry (Z) databases (EMODnet and NIB) were applied to estimate the reflectance of infinitely deep water (R_∞_), the diffuse attenuation coefficients (K_d_), and finally, bottom reflectance values (R_b_). Figure 2a indicates the location of the given polygon representing depths below 40 meters. The corresponding median values of these 43,000 R_w_ pixels were determined as 0.0200 for Band 2 (490), 0.0136 for band 3 (560) and 0.0045 for Band 4 (665). 

**Figure 3:**
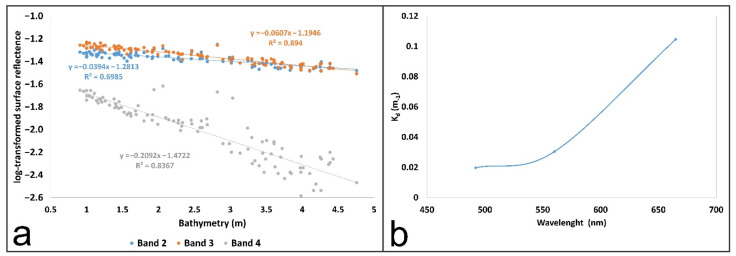
Bathymetry versus log-transformed water surface reflectance for bands 2 (R_w_ = 490), 3 (R_w_ = 560) and 4 (R_w_ = 665) (**a**) and spectral dependence of diffuse attenuation coefficient for water surface reflectance values for bands 2 (R_w_ = 490), 3 (R_w_ = 560) and 4 (R_w_ = 665) (**b**). Values of attenuation coefficient are in meters because they are depth-specific, since reflectance values are unit-less.

The NIB bathymetry database and the habitat (vegetation) type map for 2020 enabled the estimation of the diffuse attenuation coefficients (Band2 K_d_, Band 3 K_d_ and Band 4 K_d_) (Figure 2b). The first one was used to identify areas with a gradual increase in depth between 1 and 5 m, and the second one to select a non-vegetated stable substrate covered predominately with sand. The water attenuation coefficient is spectral dependent and increases with longer wavelengths (Figure 3a), converting to sensor noise in deep waters. Thus, the log-transformed surface reflectance values (R_w_) for each band in this area were plotted against the actual depth values. The resulting significant linear relations (with high R^2^ values) justify the adequate selection of the submerged sandy area for K_d_ value estimation in the study area. Moreover, they were derived from the linear regression slope coefficients, which represented the −2K_d_ parameter for each band in Equation (1). The estimated values were determined as 0.0197 for Band 2 (490), 0.0303 for band 3 (560) and 0.1046 for Band 4 (665) (Figure 3b).

### 2.3. Supervised Image Classification and Its Validation

The image pre-classification steps significantly improved the Sentinel-2 BOA product, revealing much more detail in the shallow waters where the target seagrasses thrive. The Rb composite (10-m bands 2, 3 and 4) served as the input raster for classifier algorithm training and later image classifying. In order to assure the best possible estimate of seagrass cover in 2020, three supervised classifiers were calibrated (the Maximum Likelihood Classifier (MLC), the Random Trees Classifier (RTC) and the Support Vector Machine Classifier (SVM)), and their products were compared (Table 1).

For quality control, confusion matrices for each classifier algorithm were calculated. Since misclassifications were detected in all image classifier algorithms (Table 1); the developed seagrass distribution map for year 2020 contained a certain degree of uncertainty. The best performing image classifier technique was selected by measuring the overall agreement, or level of uncertainty, in both categories (Table 1). The SVM classifier reached the highest Overall accuracy (84%) and Kappa index (0.60), followed by the MLC (Overall accuracy = 83%; Kappa = 0.59) and RTC (Overall accuracy = 81%; Kappa = 0.56) algorithms. In other words, the SVM classifier provided a seagrass distribution product for year 2020 with the lowest level of uncertainty (16%). Regardless, all classifiers predicted seagrass cover with satisfying accuracy; larger differences emerged in the category Other.

### 2.4. Seagrass Meadow Dynamics

**Figure 4:**
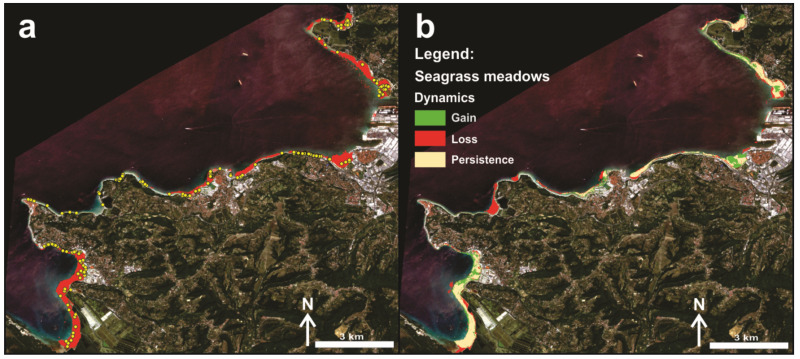
SVM classified pre-processed Sentinel-2 image showing: (1) **a**–seagrass cover in 2020 (red polygons are classified seagrasses, yellow dots are accuracy assessment points), and (2) **b**–seagrass dynamics along the Slovenian coast between 2014 and 2020.

The best performing classifier (SVM) was further applied to estimate the overall coverage of seagrass meadows in the study area for the second time window (2020) (Figure 4a). The temporal perspective indicated stable overall coverage of seagrass meadows in the study area (282.4 Ha in 2014 and 283.5 Ha in 2020). However, the spatial perspective revealed a different evolution of the current extent of seagrasses (Figure 4b). There were areas of almost complete retreat of seagrass meadows (the Strunjan Landscape Park marine area); on the other hand, there are areas of seagrass meadows that have undergone progression (the Bay of Koper and the Bay of Piran). The stable seagrass meadows part covered 191.5 Ha of area. On 90.8 Ha of sedimentary bottom, seagrass meadows have disappeared since 2014, but have recolonized 92 Ha of other areas along the Slovenian coastline.

## 3. Discussion

Marine phanerogams are considered biological sentinels or indicators because any change in seagrass distribution, such as a reduction in the maximum depth limit or widespread loss of their meadows, signals negative changes in the environment [26,47]. In recent decades, both the areas of existing seagrass beds and their losses have been very roughly estimated, while accurate data were scarce in well-studied areas, and almost completely lacking for most seas, including many Mediterranean regions [5]. The majority of available results for the Mediterranean basin are based on experts’ personal knowledge and are mainly point data on the occurrence of seagrasses [48]. The lack of such valuable information hinders any efforts to effectively manage and conserve seagrass beds in the Mediterranean Sea. However, advances in remote sensing technology and better availability of high quality (better spatial, radiometric, spectral and temporal resolution) satellite products filled the existing gap in recent years and thus enabled seagrass monitoring on the local [49,50], regional [51] and even global [52] scales. In the Mediterranean region, more studies have focused on *P. oceanica* [53,54,55,56] than on *C. nodosa* [37,57,58] or *Z. noltei* [43]. However, our results were produced by following, and modifying, methodological steps and procedures presented by Traganos and Reinartz [46], who utilized the Sentinel-2 mission for the detection of seagrass beds (both *P. oceanica* and *C. nodosa*) in the north-western Aegean Sea. In contrast, we used the BOA product and thus avoided atmospheric correction procedures. Bottom reflectance values were calibrated with own bathymetry data. Spectral signature polygons for categories seagrass and other were generated by using in situ mapping data covering the entire Slovenian coast. This application of *in situ* and remote sensing data yielded a high quality estimate of past and present seagrass cover in Slovenia. Three seagrass species, the dominant *C. nodosa*, as well as *P. oceanica* and *Z. noltei*, both less abundant in this area, were detected and mapped in the field. Namely, those three species could not be distinguished on the satellite image because *P. oceanica* and *Z. noltei* form small, fragmented patches unsuitable for training site generation from the Sentinel-2 spatial resolution perspective. This issue could potentially be solved by applying multi-temporal aerial imagery instead of satellite-based products, as demonstrated by Kaufman and Bell [59] in Tampa Bay, Florida. However, other studies proved that Sentinel-2 data could provide enough information to investigate seagrass species-specific, either long- or short-term, spatial dynamics if target species form larger patches [35,43,60,61,62]. An overall seagrass classification accuracy of 84% in our study area, for 2020, nonetheless enabled a multi-temporal spatial analysis, particularly for the *C. nodosa* meadow. 

These results highlight that the local dynamics of seagrass habitats exhibit non-linear retraction behavior (Figure 4b), despite clear regional or even global negative trends [2,3,6]. However, some important regressions were confirmed locally. In 2014, there was an extensive *C. nodosa* meadow on sandy bottom at depths of 3 to 11 m in the Bay of Strunjan (Strunjan Landscape Park). Between 2017 and 2018, the meadow shrank drastically, and there remains only a small strip in deeper parts of the bay [63]. The main factor leading to this shrinkage was the massive siltation due to construction activities in the Roja tributary, which resulted in the seagrass meadow being almost completely buried by fine sand. Saunders et al. [64] demonstrated that the relation between sediment load and habitat suitable for seagrasses is not linear and that large increases in sediment have a disproportionate negative impact on the availability of habitat suitable for marine phanerogams. Moreover, numerous signs of anoxia were found in several pits with dead *C. nodosa* leaves covered with a sulphide layer in the Bay of Strunjan, likely due to decaying seagrass and other organisms beneath the sediment or suggesting that other undiscovered factors may also be contributing to the decline of this seagrass meadow. A less remarkable decline of the *C. nodosa* meadow present in the Stjuža coastal lagoon, which is also part of the Strunjan Landscape Park, was also detected [65]. These results underscore that at the local scale, anthropogenic disturbance plays a major role in the survival of seagrass meadows. Damage to and loss of seagrass beds from human activities can be both direct-physical (burial, uprooting) and chemical (various forms of pollution), and indirect through environmental modification (like increased water turbidity and sediment deficit), as reported by Boudouresque et al. [5]. On a global scale, however, ocean acidification and warming are among the greatest threats directly or indirectly affecting seagrass survival, distribution and physiological performance [66,67,68]. For most marine macrophytes, temperature has been shown to be the most important limiting factor for dispersal and physiological activities [69,70,71]. Since sea temperature has been slowly but steadily increasing throughout the Mediterranean in recent decades, this will also affect oxygen production and respiration in *C. nodosa* meadows, as noted by Perez and Romero [72]. Therefore, future research on the long-term acclimation capacity of seagrass meadows and/or adaptation of species with thermal responses should be planned in light of climate change. Additionally, since marine phanerogams are important ecosystem builders, further research should address how associated biota (such as bacterial communities, small algae and other epibionts) may be affected and affect the adaptive plasticity of seagrasses to climate change and acidification [73,74]. Since seagrass meadows provide multiple ecosystem services per unit area, the present results related to spatio-temporal dynamics of seagrass habitats are of critical importance for the development of management and conservation plans. The establishment of effective spatial management plans aimed at mitigating the impact of human activities is of paramount importance [3] and contributes to the achievement of Good EnS on the European and Mediterranean scales. 

## 4. Materials and Methods

### 4.1. Study Area

The Gulf of Trieste is a shallow sea basin in the northernmost part of the Adriatic and the Mediterranean Sea, with an average depth of about 21 m. The seabed of the Slovenian Sea is predominantly a soft sedimentary bottom of fluvial origin, while the coastal bottom is mostly rocky and consists mainly of flysch layers of Eocene age, while in the inner bays a soft sedimentary bottom predominates [75]. It is influenced mainly by freshwater inflow, and resuspension of bottom sediments [75]. The surface sediments of the coastal zone, which is a few dozen meters wide and coincides with the 5-meter isobath, consist of silt and sandy silt, with about 30% carbonate [75]. The circulation of water masses in the Gulf of Trieste is variable but generally follows the counterclockwise circulation system of the northern Adriatic and is mainly influenced by the tidal regime (with an amplitude of about 0.5 m) and wind-driven currents [75,76]. In recent decades, the Slovenian coastal sea has suffered from many anthropogenic influences such as new infrastructure, intensive fishing, sewage discharge and mariculture [77]. Contemporary studies of decline in chlorophyll *a* concentrations throughout the northern Adriatic, consistent with declines in phosphate and ammonia concentrations [78,79], underscore the oligotrophication of the basin in recent decades [80]. 

### 4.2. Seagrass Distribution Data

#### 4.2.1. In Situ Data and Mapping

In 2014, a field-based survey of seagrass meadows along the Slovenian coast was performed by applying a high-resolution Multi Beam Sonar (MBES; Reason SeaNat 8125) and sound velocity profiler (SVP) system [81]. The MBES records 120 degrees of the seabed simultaneously, using 240 dynamically oriented beams. Seafloor detection with this sonar is extremely accurate; the depth resolution is 6 mm, and measurements can be monitored in real time on the sonar’s display. However, the final vector type (polygon) seagrass distribution map was additionally validated with air-borne imagery, national ortho-photo imagery [82] and geo-referenced underwater (horizontal (along-coast) and vertical (cross-coast)) transects recorded with an underwater camera. This product, provided by the company Harpha Sea d.o.o. (Koper, Slovenia) in 2014, served as the baseline seagrass meadow status in our study. 

In 2020, the entire Slovenian sea coast was surveyed by applying a field method based on visual observation of sea-bottom segments covered with vegetation in the infralittoral belt. The familiar CARLIT method [83,84] was improved to be suitable for the Slovenian coastal area (Figure 1), where it is not possible to apply the method to the mediolittoral belt (as defined in the original methodology), since almost all macroalgal and seagrass species living in the northern Adriatic do not grow in such shallow waters [85,86]. The survey involved a cruise along the entire coastline in a small boat, kept close to the shoreline. In the absence of littoral communities, sublittoral communities were identified using a large Aquascope Underwater Viewer and directly annotated in a graphic display. This graphic support was prepared at an appropriate scale (small enough to distinguish the shorter length of the sector being mapped) and was suitable for use in the field. The result is a division of the shoreline into several sectors, each identified by a vegetal community category (corresponding to a single community or a combination of communities). The information obtained on the distribution of community categories was then vectorized in the ArcGIS environment. The final map with all vegetation types, including seagrass meadows at 1 to 4 m depth, was considered in the following steps as (a) training and (b) validation data (along additional ground truth points in deeper waters) for supervised classification of the multi-spectral Sentinel-2 satellite image.

In waters deeper than 4 meters, additional seagrass ground control points (*n* = 50) were derived from regular diving transects in 2020 along the entire Slovenian coastline. 

#### 4.2.2. Satellite Data

In order to evaluate spatio-temporal changes in seagrass cover along the Slovenian coast, a cloud free Sentinel-2 (Level-2A, Bottom of Atmosphere (BOA)) satellite image for year 2020 (8 August 2020) was downloaded from the Copernicus Open Access H (https://scihub.copernicus.eu/dhus/#/home; accessed on 10 January 2022). Bands 2 (blue), 3 (green) and 4 (red), covering the visible spectrum (between 390 to 700 nm), were used in all further analyses, since they penetrate the water column more deeply and thus provide sensitive quantitative data on bottom reflectance [46]. 

#### 4.2.3. Auxiliary Data

To generate bottom reflectance values from the Sentinel-2 image, bathymetry data were needed. Here, Piran’s Marine Biology Station (National Institute of Biology, NIB) vector bathymetry database was used. This product is far more accurate than bathymetric estimates based on Sentinel-2 bands 2 and 3 [46,87]. However, to meet the requirements for bottom reflectance calculation [88], these records were rasterized and thus adjusted to Sentinel-2 visible bands spatial resolution (pixel size = 10 m). For calculating bottom reflectance values in the study area, a second bathymetric dataset (horizontal resolution of 100 m), which covered the whole area of the satellite image, was downloaded from the EMODnet web platform (https://portal.emodnet-bathymetry.eu/; accessed on 10 January 2022) (Sector E6 2020). 

To focus only on the shallow coastal part of the sea, where seagrass meadows are commonly present, a satellite image mask had to be applied. This is an important step in coastal remote sensing, owing to the enhancement of coastal water features by removing all terrestrial features. We clipped out the mainland with the national coastline vector database [82] provided by the national Surveying and Mapping Authority, which operates under the Ministry of the Environment and Spatial Planning. In addition to that, the bathymetric contour of 20 m depth was used to clip the initial satellite image granule to the coastal study area, masking out deeper waters (more than 20 m) where reliable quantitative estimates of the seafloor are difficult to derive [46].

### 4.3. Water Column Correction

The water column represents noise in optical remote sensing products when they are used to investigate traits of submerged habitats. From that perspective, its effect requires correction. By following the methodological approach described in Maritorena et al. [88] and further applied in Traganos and Reinartz [46], the water column correction was based on an analytical model for optically shallow water. The algorithm equals the bottom of atmosphere surface reflectance values (R_w_) to the reflectance of an infinitely deep-water column (R_∞_), to which a substrate contrast was added (bottom reflectance, R_b_ −R_∞_) after correction of the water depth effect (the term e^(−2KdZ)^ where K_d_ is the light attenuation coefficient in the water and Z represents water depth).

R_w_ = R_∞_ + (R_b_−R_∞_)e^(−2KdZ)^ (1)

Thus, pixel level bottom reflectance can be calculated from values of R_w_, estimation of R_∞_, values of Z and estimate of K_d_.

#### 4.3.1. Infinitely Deep-Water Reflectance Estimation

According to the Maritorena et al. [88] algorithm (1), the necessary deep-water reflectance parameter R_∞_ was derived from the water surface reflectance values of the deepest part of the sea captured by the obtained Sentinel-2 image. We identified these deep waters (pixels below 40 m depth (an area of 4.3 km^2^), where reflectance values are not, or just slightly, affected by the bottom signal) by using the free available EMODnet bathymetry data (sector E6 2020) (https://portal.emodnet-bathymetry.eu/; accessed on 10 January 2022), which covered the whole area of the satellite image (Figure 2A). Thus, a median R_w_ value of the 4.3 km^2^ area was calculated for each considered band (2, 3 and 4) of the satellite image.

#### 4.3.2. Diffuse Attenuation Coefficient Estimation

In the next image pre-processing step, the transparency properties of water in the study area were evaluated by calculating the diffuse attenuation coefficient K_d_ values for each considered Sentinel-2 band (2, 3 and 4), according to Bierwirth et al. [89]. A uniform sandy area stretching from 1 to 5 m in depth, as proposed by Maritorena et al. [88], was selected to get functional relations between bathymetry and log-transformed surface reflectance values (R_w_) for bands 2, 3 and 4 (Figure 3). Thus, K_d_ values were estimated using the slope of each regression line, which represents the quantity −2K_d_ for each band [35,89]. 

### 4.4. Image Classification 

After obtaining all the variables needed for equation 1 (R_∞_, Z and K_d_), bottom reflectance values (R_b_) for each visual band of the satellite image were calculated. The final R_b_ composite was then the source for supervised image classification techniques supported by training polygons determined by field mapping in 2020 and additional diving-based ground control points (*n* = 50) in deeper waters (4 to 11 m in depth). However, two categories were distinguished: seagrasses and others. The latter is a mosaic of several habitat types related to the varied type and structure of the seafloor substrate along the Slovenian coast. This includes non-vegetated sandy areas and rocky bottoms covered with various vegetation types, such as canopy-forming species (mainly *Gongolaria barbata*, *Cystoseira compressa* and *Halopithys incurva*) belts and/or lower vegetation cover (*Padina pavonica*, *Dictyota* spp., *Halopteris scoparia*, *Corallinaceae*, etc.) We aggregated all these habitats because they mostly form small fragmented (dislocated) patches unsuitable for training polygon creation in the study area from the Sentinel-2 spatial resolution perspective (pixel size = 10 m). Thus, the ground control sample was divided into training (40%) and accuracy assessment data (60%) for both categories (seagrass and other) for all selected supervised classifiers, as suggested by Congalton [90] and Green et al. [91]. Here, three algorithms in the ArcGIS environment [92] were applied: (1) the Maximum Likelihood Classifier (MLC), (2) the Random Trees Classifier (RTC) and (3) the Support Vector Machine Classifier (SVM). 

### 4.5. Accuracy Assessment

Quality control is essential in remote sensing products. Quantifying the amount of error in a classified image is thus crucial to reach the best link between image and reality. From that perspective, a confusion matrix with errors of omission and commission for each image classification algorithm (MLC, RTC and SVM) was computed in the ArcGIS environment [92], based on accuracy assessment points derived from seagrass meadow ground control points (field mapping-and diving-based) considered as accuracy assessment data (60%, as stated in Section 2.3). The best performing seagrass classification algorithm was then selected for image change analysis in the next step.

### 4.6. Change Analysis

The remote sensing part of this study was performed with the clear purpose of evaluating the spatio-temporal dynamics of seagrass meadows along the Slovenian coast. We tested the assumption that these important ecosystem engineers have been losing ground in recent decades, at either the local (Adriatic Sea), regional (Mediterranean Sea) or even the global level. We compared two time windows (2014 and 2020) in which field-based mapping was performed, to secure the highest possible accuracy of seagrass meadow distribution in the study area. By overlaying the sonar-based baseline status from 2014 with the Sentinel-2 classified product for 2020, three processes were identified. First, temporally and spatially stable seagrass habitats; second, temporally and spatially dynamic areas, where seagrass meadows either progressed or disappeared. However, since the baseline seagrass cover status in 2014 was compared against a partial remote sensing product with existing misclassifications in 2020, the change analysis should be considered as an estimation with a certain level of uncertainty (16% for the selected SWM classifier). 

## 5. Conclusions

The integration of field-based and remote sensing methods (the Sentinel-2 mission) allows fine-scale mapping and monitoring of interannual and also decadal changes in seagrass distribution, from the small scale to the broader one. If systemized, this approach to monitoring seagrass meadow dynamics could be developed as a spatial decision support system for the entire Mediterranean basin. The resulting spatio-temporal change maps of seagrass meadows would serve as a key tool for decision makers in marine protected areas and would potentially support more effective conservation and management actions in these highly productive and important environments. Moreover, key environmental change predictor variables could be added to the decision support system, as well as climate change predictions, to generate potential seagrass meadow risk maps. However, there are some limitations in such an approach. For sure, there is some bias in surface cover estimation since temporal comparisons need to carefully be done for the same co-registered area. Nonetheless, such an information system could help in planning large scale restoration actions which are urgently needed to cope with environmental problems linked, directly or indirectly, to the deepening climate crisis. 

## Figures and Tables

**Table 1:** Confusion matrix summary.

**SVM**	**Seagrass**	**Other**	**Total**	**User Accuracy**
Seagrass	324	21	345	0.94
Other	60	97	157	0.62
Total	384	118	502	
Producer Accuracy	0.8420	0.82		
Overall Accuracy	0.84			
Kappa	0.60			
**MLC**	**Seagrass**	**Other**	**Total**	**User Accuracy**
Seagrass	319	20	339	0.94
Other	63	97	160	0.61
Total	382	117	499	
Producer Accuracy	0.83	0.83		
Overall Accuracy	0.83			
Kappa	0.59			
**RTC**	**Seagrass**	**Other**	**Total**	**User Accuracy**
Seagrass	303	15	318	0.95
Other	79	102	181	0.56
Total	382	117	499	
Producer Accuracy	0.79	0.87		
Overall Accuracy	0.81			
Kappa	0.56			
Kappa	0.56			

## Data Availability

Data sharing not applicable.
